# P-316. Comparison of At-Home Versus In-Clinic Administration of Long-Acting Injectable Cabotegravir for Pre-Exposure Prophylaxis (PrEP)

**DOI:** 10.1093/ofid/ofaf695.535

**Published:** 2026-01-11

**Authors:** Michael A Garber, Rebecca Wittenberg, Shelly Willis, Kofi Andoh, Joy Vongspanich Dray, Selina Somani, Sarah Waldman

**Affiliations:** UC Davis Health, Sacramento, CA; UC Davis, Sacramento, CA; UC Davis Health, Sacramento, CA; UC Davis Health, Sacramento, CA; UC Davis Health, Sacramento, CA; UC Davis Health, Sacramento, CA; University of California Davis, Sacramento, CA

## Abstract

**Background:**

Numerous barriers exist related to provision of long-acting (LA) injectable antiretroviral therapy (ART) services in-clinic. The in-home administration of LA ART has been explored for human immunodeficiency virus (HIV) treatment and was found to be favorable to address several challenges.1 There is strong interest in similarly exploring in-home services for cabotegravir (CAB) LA for HIV pre-exposure prophylaxis (PrEP).2

**Methods:**

Persons prescribed CAB-LA in the Infectious Diseases clinic at UC Davis Health were considered for enrollment in this non-randomized, observational analysis between June 2024 and April 2025. Eligible persons (per insurance coverage) were offered either in-clinic or in-home (via home health) services for receipt of CAB LA with the option to later transition to an alternative site of care if desired.

**Results:**

Persons (n = 43) who were prescribed CAB LA during the analysis period were largely male (88.4%), cis gender (83.7%), and commercially insured (81.4%) with a median age of 38 and nearly half (46.5%) belonging to a racial minority group. Among those prescribed CAB LA, six (13.9%) had not yet started and were excluded from this analysis. Of the 37 (86.1%) who began CAB LA, 18 (48.6%) were deemed eligible for in-home services based on insurance. A total of nine (24.3%) received in-home services: two (5.4%) initiated CAB LA in-home and seven (18.9%) transitioned from clinic to home administration. The remaining 28 (75.7%) initiated and continued CAB LA in clinic. An average of 3.85 in-home versus 5.9 in-clinic visits were completed per patient, with zero participants in-home and five (14.3%) in-clinic receiving off schedule dose(s). Regardless of site of care, all patients remained HIV seronegative with no medication safety concerns and only two (5.4%) participants were lost to follow-up.

**Conclusion:**

The in-home administration of CAB LA by a healthcare practitioner is feasible, comparably safe, effective, and possibly preferred to in-clinic administration among participants.

**Disclosures:**

Sarah Waldman, MD, Merck Parmaceuticals: Grant/Research Support

